# Hepatitis B Virus Variants with Multiple Insertions and/or Deletions in the X Open Reading Frame 3′ End: Common Members of Viral Quasispecies in Chronic Hepatitis B Patients

**DOI:** 10.3390/biomedicines10051194

**Published:** 2022-05-21

**Authors:** Selene García-García, Andrea Caballero-Garralda, David Tabernero, Maria Francesca Cortese, Josep Gregori, Francisco Rodriguez-Algarra, Josep Quer, Mar Riveiro-Barciela, Maria Homs, Ariadna Rando-Segura, Beatriz Pacin-Ruiz, Marta Vila, Roser Ferrer-Costa, Tomas Pumarola, Maria Buti, Francisco Rodriguez-Frias

**Affiliations:** 1 Liver Pathology Unit, Departments of Biochemistry and Microbiology, Vall d’Hebron University Hospital, 08035 Barcelona, Spain; selene.garcia@vhir.org (S.G.-G.); acaballero@laboratorioechevarne.com (A.C.-G.); a.rando@vhebron.net (A.R.-S.); beatriz.pacin@vhir.org (B.P.-R.); marta.vila.salvador@vhir.org (M.V.); roferrer@vhebron.net (R.F.-C.); 2 Centro de Investigación Biomédica en Red de Enfermedades Hepáticas y Digestivas (CIBERehd), Instituto de Salud Carlos III, 28029 Madrid, Spain; josep.gregori@gmail.com (J.G.); josep.quer@vhir.org (J.Q.); mmriveir@vhebron.net (M.R.-B.); mbuti@vhebron.net (M.B.); 3 Biochemistry and Molecular Biology Department, Universitat Autònoma de Barcelona (UAB), 08193 Bellaterra, Spain; 4 Echevarne Laboratory, Department of Biochemistry, 08037 Barcelona, Spain; 5 Liver Diseases-Viral Hepatitis, Liver Unit, Vall d’Hebron Institute of Research (VHIR), Vall d’Hebron University Hospital, 08035 Barcelona, Spain; 6 Blizard Institute, Barts and the London School of Medicine and Dentistry, Queen Mary University of London, London E1 2AT, UK; f.rodriguez-algarra@qmul.ac.uk; 7 Liver Unit, Department of Internal Medicine, Vall d’Hebron University Hospital, 08035 Barcelona, Spain; 8 Anoia Primary Care Service, Territorial Management of Central Catalonia, Catalan Institute of Health, 08700 Igualada, Spain; mhoms.cc.ics@gencat.cat; 9 Health Promotion in Rural Areas Research Group, Territorial Management of Central Catalonia, Catalan Institute of Health, 08272 Sant Fruitós de Bages, Spain; 10 Department of Microbiology, Vall d’Hebron University Hospital, 08035 Barcelona, Spain; tpumarola@vhebron.net

**Keywords:** hepatitis B virus, hepatitis B X open reading frame, *HBX* 3′ end region, insertions, deletions, quasispecies, next-generation sequencing

## Abstract

Deletions in the 3′ end region of the hepatitis B virus (HBV) X open reading frame (*HBX*) may affect the core promoter (Cp) and have been frequently associated with hepatocellular carcinoma (HCC). The aim of this study was to investigate the presence of variants with deletions and/or insertions (Indels) in this region in the quasispecies of 50 chronic hepatitis B (CHB) patients without HCC. We identified 103 different Indels in 47 (94%) patients, in a median of 3.4% of their reads (IQR, 1.3–8.4%), and 25% (IQR, 13.1–40.7%) of unique sequences identified in each quasispecies (haplotypes). Of those Indels, 101 (98.1%) caused 44 different altered stop codons, the most commonly observed were at positions 128, 129, 135, and 362 (putative position). Moreover, 39 (37.9%) Indels altered the TATA-like box (TA) sequences of Cp; the most commonly observed caused TA2 + TA3 fusion, creating a new putative canonical TATA box. Four (8%) patients developed negative clinical outcomes after a median follow-up of 9.4 (8.7–12) years. In conclusion, we observed variants with Indels in the *HBX* 3′ end in the vast majority of our CHB patients, some of them encoding alternative versions of HBx with potential functional roles, and/or alterations in the regulation of transcription.

## 1. Introduction

Hepatitis B remains a major global health problem. This infectious disease is estimated to have caused 820,000 deaths in 2019, mostly from cirrhosis and hepatocellular carcinoma (HCC), while 296 million people were chronically infected with hepatitis B virus (HBV) [1]. Neither of the current antiviral therapies (nucleoside/nucleotide analogs [NAs] and pegylated interferon alpha [IFN]) is able to achieve a complete cure of the infection. This is mainly due to the persistence of the HBV genome in the nuclei of infected hepatocytes [2], both as an episomal chromosome-like structure called covalently closed circular DNA (cccDNA) and integrated into the host’s genome. Both cccDNA and integrated forms of the viral genome are the sources of all HBV transcripts [3,4]. The HBV genome is about 3.2 Kb in length and, due to this small size, in the same nucleotide (nt) sequence, it contains four highly overlapped open reading frames (ORFs), the regulatory elements to control their transcription, and structural elements essential for viral replication [5]. The packing of information is notable in the X ORF (*HBX*, nt 1374–1838), especially in its 3′ end region (nt 1523–1838), a sequence that also contains the Core promoter (Cp), which controls the transcription of pregenomic RNA (pgRNA), an intermediate in viral replication that is also translated to HBcAg and the viral polymerase, and pre-core RNA (pcRNA), which is translated to HBeAg. This promoter includes sequence and structural motifs such as the four TATA-like boxes (TA) 1 to 4, the enhancer II (ENHII), and the direct repeat 1 (DR1), all essential for viral replication [6,7].

The *HBX* gene encodes the HBV X protein (HBx), a 17-kilodalton (KDa) protein composed of 154 amino acids (aa) [8]. This protein is characterized by an astonishing pleiotropic function thanks to its direct interactions with multiple cellular proteins. Specifically, HBx is capable of promoting HBV replication by epigenetic stimulation of cccDNA transcription [3]. Moreover, it is able to facilitate the interaction of stimulating cellular transcriptional factors to this episome, in order to regulate the transcription of host genes, disrupt protein degradation, modulate signaling pathways, manipulate cell death, and deregulate the cell cycle [9]. The HBx C-terminal end plays a key role in controlling these functions owing to its transactivating activity [10]. This region of HBx is encoded by the *HBX* 3′ end region, where deletions (Del) have been more frequently detected in the tumor than in the adjacent non-tumor tissues from HCC patients by population sequencing [11]. These Del, along with insertions (Ins) in this region of *HBX* may yield significantly altered HBx due to *HBX* frameshifts. HBx with truncations in the C-terminal end (hereinafter referred to as HBxCtermTrunc) has been associated with a critical role in HCC carcinogenesis [12]. In addition, these Indels may affect not only HBx but also the properties of the multiple important regulatory and structural motifs (Cp, ENHII, DR1, etc.), overlapped with it. Viral populations (variants) with insertions and/or deletions (Indels) contribute to the high genetic variability of HBV, which leads to a highly heterogeneous viral infection distributed as a quasispecies: a mixture or swarm of variants genetically closely related but not identical [13]. Interestingly, previous studies using clone sequencing identified variants with Indels in the *HBX* 3′ end region, both in patients with severe liver disease [14,15] and in chronic hepatitis B (CHB) patients without HCC [16,17]. This suggests that these variants are usually present in HBV quasispecies, in both severe and mild forms of liver disease, despite the possible alterations of the HBx caused by Indels in the *HBX* 3′ region, or the effects on the promoter activity of Cp. Many of these variants may even play some functional role.

The aim of this study was to investigate the presence of variants with Indels in the *HBX* 3′ end region in CHB patients without HCC using next-generation sequencing (NGS). This high-throughput technology can reveal these kinds of variants even if they are present as minor variants in the quasispecies, undetectable using Sanger or clone sequencing. We identified variants with Indels in the *HBX* 3′ end region in most of our CHB patients, and potential alterations to HBx and Cp and functional consequences of the most relevant of those Indels have been discussed.

## 2. Materials and Methods

### 2.1. Patients and Samples

In this retrospective study, CHB patients who attended the outpatient clinic of Vall d’Hebron University Hospital (Barcelona, Spain) were selected according to the following inclusion criteria: age over 18 years; available serum sample with HBV-DNA levels of 1000 IU/mL or higher (to ensure sufficient HBV-DNA levels to study their quasispecies) obtained after more than 6 months with detectable HBsAg, in a period without antiviral treatment and no evidence of HCC; negative test for hepatitis C virus (HCV), human immunodeficiency virus (HIV), and hepatitis D virus (HDV) infections; written informed consent for participation provided.

### 2.2. Serological and Virological Determinations

Serological markers of HBV and HCV infections [HBsAg, HBeAg, and antibodies against HCV (anti-HCV)] were tested using commercial electrochemiluminescence immunoassays on a COBAS 8000 instrument (Roche Diagnostics, Rotkreuz, Switzerland). Anti-HDV antibodies were tested using the HDV Ab kit (Dia.Pro Diagnostics Bioprobes, Sesto San Giovanni, Italy), and anti-HIV antibodies with the Liaison XL murex HIV Ab/Ag kit (DiaSorin, Saluggia, Italy). HBV-DNA was measured in the Cobas 6800 System (Roche Diagnostics, Mannheim, Germany), with a detection limit of 10 IU/mL. HBV genotyping was performed using a line-probe assay (INNO-LiPA HBV Genotyping Assay; Fujirebio Europe N.V., Ghent, Belgium).

### 2.3. Amplification of HBV Genome Region Analyzed and Next-Generation Sequencing

In this study, a fragment of the HBV genome located between nt 1596 and 1912 was amplified using an in-house nested PCR and sequenced on forward and reverse strands by means of NGS, using ultra-deep pyrosequencing (UDPS) technology [Genome Sequencer FLX and Junior systems (454 Life Sciences-Roche, Branford, CT, USA)], as previously described [18]. Briefly, HBV-DNA was extracted from 200 μL of serum using QIAamp DNA Mini Kit (QIAGEN, Hilden, Germany) as per the manufacturer’s instructions. Nested PCRs were performed using the PfuUltra II Fusion HS DNA polymerase (Agilent technologies, Santa Clara, CA, USA), using the following primers: outer PCR, forward 5′-*GTTGTAAAACGACGGCCAGT*TGTGCACTTCGCYTCACC-3′ and reverse 5′-*CACAGGAAACAGCTATGACC*AGWAGCTCCAAATTCTTTATAAGG-3′. These primers contain an M13 universal adaptor sequence at the 5′ end (in italics) and the template-specific sequence. The nested PCR primers contained 5′ 25-nt sequences A and B, which are adaptors for the elements of the 454 Life Sciences-Roche UDPS system, followed by a 10-nt sequence used as a unique identifier for each sample (multiplex identifier), and the same M13 universal adaptor sequences included in the outer PCR primers at the 3′ ends. Finally, 468 base pairs (bp) amplicons were obtained, which were pooled and processed according to the 454 Life Sciences-Roche UDPS protocol.

The NGS raw data presented in this study are openly available in the NCBI database Sequence Read Archive (SRA), at BioProject accession number PRJNA625435. The BioSample accession numbers are included in Table S1.

### 2.4. Next-Generation Sequencing Data Treatment

The HBV genomic fragment analyzed was sequenced on both strands, forward and reverse. The sequences obtained by NGS (referred to as reads) were processed using a data treatment workflow, established in previous studies with HBV [19] and HCV controls [20] to minimize the scoring of PCR artifacts and sequencing errors: the reads obtained were demultiplexed by identifying the multiplex identifier and the template specific primer sequences. Primers were then trimmed and both strands were treated separately. First, the reverse reads were reverse complemented, and all (forward and reverse) reads that (1) did not cover the full amplicon, (2) had more than one indetermination, and (3) had an identity relative to the reference sequence below 70% were discarded. The resulting sequences were collapsed to haplotypes (HPL), i.e., unique sequences covering the full amplicon with their corresponding frequencies, thus each HPL corresponds to a quasispecies variant.

Multiple alignments of the forward and reverse HPL of each sample, with abundances not below 0.1%, were then performed with a reference sequence of the corresponding genotype (Table S2), using the MUSCLE software package (version 3.8.31) [21]. Only those HPL common to both strands were retained, and those unique to a single strand were removed. The resulting HPLs were called consensus HPLs and their final frequencies were taken as the sum of the read counts in each strand. The multiple alignments of the consensus HPLs with the corresponding reference sequence were used to report Indels.

All computations were performed with the R environment and language [22], using in-house scripts with the help of the Biostrings [23] and ape packages [24].

### 2.5. Cloning to Confirm NGS Results

The presence of variants with Indels identified in this study was confirmed by cloning and sequencing of some selected samples. The same PCR products obtained from these samples and analyzed by NGS were also cloned using Zero Blunt TOPO PCR cloning Kit (Life Technologies, Carlsbad, CA, USA), following the manufacturer’s instructions. Briefly, the 468-bp amplicons obtained using nested PCR were cloned into pCR™4Blunt-TOPO^®^ vector and the resulting constructs were used to chemically transform *Escherichia coli* competent cells using the heat shock method. The transformed bacteria were incubated on a Luria–Bertani (LB) broth agar plate overnight at 37 °C and 18–29 clones/samples were selected for sequencing.

In those selected clones, the pCR™4Blunt-TOPO^®^ vectors containing a 468-bp amplicon sequence were isolated with the QIAprep Miniprep kit (QIAGEN, Hilden, Germany), following the manufacturer’s instructions. Finally, the amplicon sequences were directly sequenced using the BigDye Terminator v3.1 Cycle Sequencing Kit (Thermo Fisher Scientific, Waltham, MA, USA), with the primers M13-Fw and TOPO Rv provided in the Zero Blunt TOPO PCR cloning Kit, on an ABI PRISM 3130xl Genetic Analyzer (Thermo Fisher Scientific, Waltham, MA, USA).

### 2.6. RNA Structural Modelling

The structure of significant sequence motifs included in the analyzed region, with or without Indels identified, was analyzed at the theoretical level. To this end, the local RNA structures obtained from the sequence of selected HPL were modeled using the RNAfold Webserver (http://rna.tbi.univie.ac.at/cgi-bin/RNAWebSuite/RNAfold.cgi (accessed on 28 March 2022)), from the Vienna RNA Websuite [25]. The Vienna RNA WebServers are based on the latest Vienna RNA Package (Version 2.4.18). All parameters of the web application were set to default. Secondary RNA structure was predicted using generalized centroid estimators.

### 2.7. Statistical Analysis

All statistical analyses were performed in R [26]. Proportions of cases showing a specific Indel have been compared between HBeAg-positive and negative patients using Fisher’s exact test. The percentages of reads and HPL with Indels were shown as median and interquartile range (IQR). Statistical comparisons between median percentages of reads and HPL with Indels in HBeAg-positive and HBeAg-negative patients were performed using the Kruskal–Wallis test. Correlation of these median percentages with HBV-DNA levels were assessed by Spearman’s correlation coefficient. P values of less than 0.05 were considered significant.

## 3. Results

### 3.1. Patients and Samples

A group of 50 CHB patients was selected, most of whom were HBeAg-negative and did not show a significant fibrosis degree (≤F3). Demographical, clinical, virological, serological, and biochemical markers from the patients included in this study are summarized in Table 1.

### 3.2. Overview of Next-Generation Data Analyses

**Figure 1:**
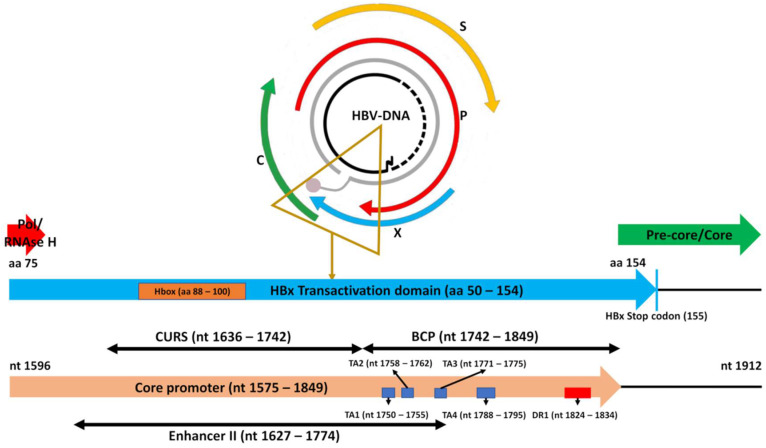
Fragment of hepatitis B virus (HBV) genome analyzed in this study. It encompasses parts of the Polymerase, X (*HBX*), and pre-core/core open reading frames (ORF). The fragment of *HBX* analyzed encodes most of hepatitis B X protein (HBx) transactivating C-terminal domain, including its essential α-helical motif (Hbox) [27]. In addition, the nucleotide (nt) sequence of that fragment (nts 1596 to 1912) contains most of the core promoter sequence, including the core upstream regulatory sequence (CURS) and the basic core promoter (BCP) with the TATA-like boxes 1–4 (TA1–TA4); nt positions are shown as described in [6]. It also includes the Enhancer II and the direct repeat 1 (DR1) sequences; nt positions are shown as described in [7].

The fragment of the HBV genome between nt 1596 and 1912 encodes from aa 75 to 154 of HBx, along with the last 8 aa of the RNAse H in the polymerase ORF and the pre-core and the first 5 aa of the core region in the pre-core/core ORF (Figure 1). In this study, we focused on the effects of Indels on *HBX* and the overlapped Cp; their effects on these short polymerase and core ORFs stretches were considered beyond the scope of this study and were not explored.

NGS and bioinformatics processing of the amplicon libraries of this fragment of HBV genome, obtained from the 50 included samples yielded a total of 960,921 reads, a median of 16,735/sample (IQR, 9277–24,668). Of those 960,921 reads, 70,278 (7.3%) showed Indels, including single Ins, Del, or combinations of them, affecting the HBx coding sequence. Interestingly, those reads were found in 47/50 (94%) patients, mainly in small percentages (median of 3.4% per sample [IQR, 1.3–8.4%]). HBeAg-positive patients showed higher percentages of Indel reads than HBeAg-negative patients (median of 6.1% versus [vs.] 1.5%, respectively; *p* = 0.03). The percentage of reads with Indels did not show any statistically significant correlation with HBV-DNA levels, whether considering all patients together or separating them according to HBeAg status.

Those reads were grouped in a total of 1039 HPL (median of 17/sample [IQR, 11–28]), of which 288 (27.7%) showed Indels and represented a median of 25% (IQR, 13.1–40.7%) of HPL found in each sample. Among the Indels identified in those 288 HPL, 103 different single Ins or Del, or combinations of them affected the HBx coding region (named as ID: 1–103, Table S3). While median percentages of Indel HPL were higher in HBeAg-positive patients than in HBeAg-negative (33.3% vs. 25%, respectively), the differences were not statistically significant. The correlation between Indel HPL and HBV-DNA levels was also not statistically significant.

Of those Indels, 12 (11.7%) were observed in at least 10% of patients (Table 2). Of these Indels, ID: 11 and 30 were only present in HBeAg-negative patients; however, the percentages of cases showing these Indels showed no statistically significant differences between HBeAg-positive and HBeAg-negative patients. ID: 85, on the other hand, showed a lower median percentage of reads in HBeAg-positive patients than in HBeAg-negative patients (0.4% vs. 1.1%, respectively; *p* = 0.01), although no statistical difference in terms of proportion of positive cases had been observed. No other statistically significant differences between HBeAg-positive and HBeAg-negative patients were observed in this group of Indels. No significant correlation was observed with HBV-DNA levels.

### 3.3. Alternative HBX Stop Codons

Almost all the different Indels identified (101/103, 98.1%) altered the position of the wild-type (WT) *HBX* stop codon 155 (Table S3). These 101 Indels were detected in all 47 patients showing Indel reads, causing 44 different altered stop codons, 15 (34.1%) of which were identified in more than 10% of patients (Table 3). Of these codons 23 (52.3%) were located before the WT position, leading to a HBxCtermTrunc, while 21 (47.7%) were located beyond the WT position, leading to an elongated HBx (HBxLong).

The most frequent premature stop codons among patients were those in positions 128, 129, and 135, associated with several different Indels (Table 3). Interestingly, while the premature stop codon 128 was associated with single nt Ins in positions 1739, 1746, and 1751 (ID: 37, 38, and 41, respectively), stop codon 129 was associated with 9 different single nt Dels between positions 1630–1749 (Table S3). Therefore, both stop codons were associated with single nt Ins or Del in a region just before TA region, or inside TA1 (Figure 1). On the other hand, stop codon 135 was associated with 13 different Indels affecting the TA region, as discussed below (Section 3.4. Indels Affecting the TATA-Like Box Region).

In relation to the stop codons beyond the WT position, it must be borne in mind that the fragment of HBV genome analyzed made it possible to continue translation in the same ORF as *HBX* until codon 179, which allowed us to identify 9 stop codons between positions 155 and 179 (in codons 156, 157, 158, 173, 174, 175 176, 177 and 179). However, some HPLs with Indels did not show any stop codon. In those cases, a reference HBV genome sequence of the same genotype was added after nt 1912, and translation was continued in the same ORF until the appearance of a stop codon. The accession numbers of reference sequences used were V01460 (for genotype D HPL) and X02763 (for genotype A HPL); no genotype F HPL showed stop codons beyond 179. Thanks to this, 12 additional putative stop codons have been identified (in codons 180, 181, 182, 183, 207, 355, 356, 357, 360, 361, 362 and 363). The putative stop codon located at position 362 was the most frequently altered stop codon identified and was observed in almost half of the patients (Table 3). Interestingly, the elongation of *HBX* to codon 362 shifted this reading frame to the core ORF, thus resulting in a putative fusion protein, which would include from aa 1 to 149 or 150 of HBx plus aa 3 or 4 to 214 of the pre-core protein. This stop codon was associated with single nt Ins located within or adjacent to a five T region (1821–1825), partially overlapped with the DR1 motive. Of note, one of these Ins was the ID: 84 (Ins 1825T), the most prevalent in patients of the 103 different Indels identified (Table 2), found in 13049/960921 (1.4%) and 23/1039 (2.2%) of total reads and HPL obtained, respectively. However, the Ins ID: 84 (and also ID: 74) are not strictly associated with the stop codon before position 362; a few HPL with both Ins showed a stop codon in position 175 (Table S3). In fact, HPL with a putative stop codon between positions 355 to 363, potentially encoding an HBx + pre-core fusion protein, were identified in 25/50 (50%) patients, in a median of 1.6% (1.2–3%) of their reads and 7.7% (4.3–14.3%) of their HPL. These putative stop codons were associated with Dels between positions 1805 and 1843 (ID: 64, 67, 69, 70, 73, 83, 92, 94, and 97), and Ins within or very close to the five T region between positions 1821–1825 (ID: 20, 74, 77, 78, 84 and 88) (Table S3).

**Figure 2:**
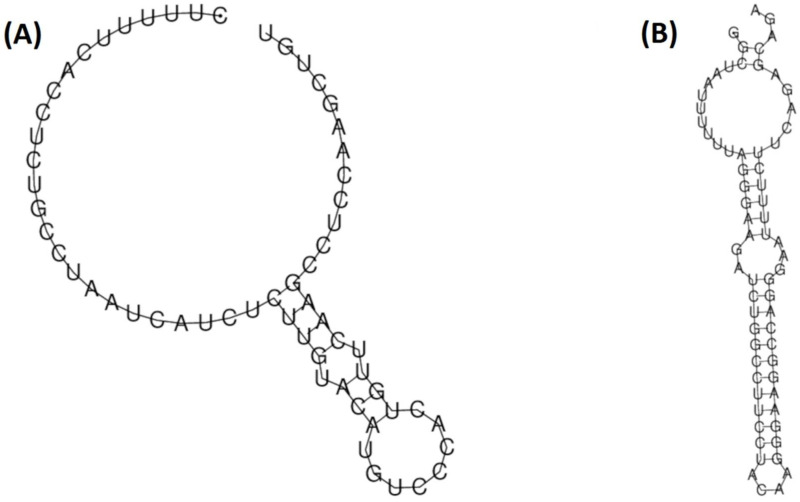
Comparison between the predicted local RNA folding of the region between nucleotides 1820 and 1880 of the hepatitis B virus (HBV) genome, including the polyT homopolymeric region between positions 1821–1825 (**A**); the region between nucleotides 1625 to 1691 of the human immunodeficiency virus-1 (HIV), including the Programmed −1 ribosomal frameshifting mRNA signal (**B**). The HBV sequence has been modeled from the genotype A reference sequence used to report Indels, included in Table S2. The HIV sequence has been modeled from the Genbank pattern with accession number NC_001802.1.

Interestingly, the Ins events in this polyT homopolymeric region would have similar consequences to the programmed −1 ribosomal frameshifting (PRF) mRNA signals. These signals are used by many viruses, such as Rous sarcoma virus and HIV-1, to induce a proportion of translating ribosomes to slip back by 1 nt into an overlapping ORF and to continue the translation, thus producing coordinated expression of two or more proteins from a single mRNA [28,29,30]. Of note, the region involving this polyT homopolymeric region in HBV showed RNA folding highly similar to that predicted for the HIV-1 PRF signal, which includes a heptanucleotide slippery sequence (UUUUUUA) followed by a spacer region and a downstream RNA stem-loop structure [29] (Figure 2). Additionally, of note in the same five T region is the single nt Del 1825 (ID: 85), present in 20% of patients (Table 2) and associated with a stop codon at position 179 (Table 3).

### 3.4. Indels Affecting the TATA-Like Box Region

A group of 39/103 (37.9%) identified Indels were located in the TA region (Figure 1). Altogether, these Indels were found in 28,628/960,921 (3%) of total reads obtained, which were grouped in 111/1039 (10.7%) HPL, and were present in 25/50 (50%) patients. Notably, these Indels were included in the region between nt 1751 and 1787; none of them affected the TA4 sequence (nt 1788–1795). The effects of these 39 Indels on the TA region are summarized in Table 4.

All Indels affecting the TA region also altered the WT *HBX* stop codon, showing 17 different stop codons, 12 (70.6%) of which led to HBxLong (Table S4). Of note among them is the stop codon in position 135, which was caused by 13/39 (33.3%) of those Indels. Of those 13 Indels, 10 (76.9%) contained 8 nt deletions (Del8nt) between positions 1757 and 1773, which eliminated TA2, caused the fusion of TA2 + TA3, or partially eliminated TA3. The stop codon in position 135 was also associated with 3 Indel combinations including Del 1758–1777, which eliminated TA2 + TA3 (Table S4). Altogether, these 13 Indels causing stop codon 135 were identified in 14 patients, 56% of 25 patients with Indels in the TA region, and 28% of the 50 patients included. These patients showed a relatively high median percentage of reads with this stop codon compared to the other stop codons associated with Indels (Table 3), even reaching 20.8% of reads obtained in a single case.

Of the 39 Indels affecting the TA region, we identified 14 (35.9% ID: 9, 12–20, 22, 24, 26, and 27) complex Indel combinations, present in 4 patients, 16% of 25 patients with Indels in the TA region, and 8% of 50 patients included. These combinations included Del which, in most cases, caused a total or partial elimination of TA2 + TA3, linked to additional big Ins (24–28 nt) in a region of the core upstream regulatory sequence (CURS) (Table S4). The CURS contains several sequence motifs that positively regulate the activity of the basal core promoter (BCP), which includes the TA (Figure 1). Notably, most of these variants contained a duplication in the motif known as α box (nts 1646–1668) [6], overlapping the *HBX* region encoding an α-helical motif (Hbox, between aa 88–100), which binds to the DNA damage-binding protein 1 (DDB1) [27], an essential interaction for HBV replication [31]. It is worth mentioning that, in a single patient, 72.9% of reads and 65.5% of HPL showed these complex Indel combinations. In particular, more than a half (51.9%) of reads obtained corresponded to Indel combination ID: 12, constituted by the 24 nt Ins 1647TCTTACATAAGAGGACTCTTGGAC, and Del 1627 + 1758 − 1777 which eliminates TA2 + TA3. Ins 1647 is a duplication of the contiguous sequence which causes the duplication of regulatory motifs in Cp such as the α box and binding sites for CCAAT/enhancer-binding protein α (C/EBPα) [6,32]. This duplication also results in a strong modification of the HBx Hbox (88SCPRSYIRGLLDS100 instead of 88ILPKVLHKRTLGLP100) [27].

**Figure 3:**
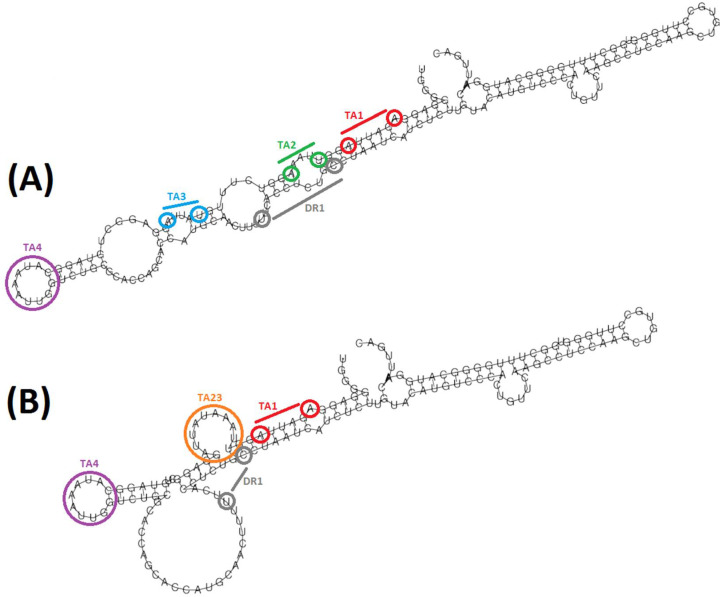
Comparison between the predicted local RNA folding between nucleotides 1741 and 1912 encompassing the TA region of the core promoter, of a WT variant (**A**) versus a variant including the deletion 1763–1770 (ID: 51) (**B**). Both variants have been modeled from the genotype A reference sequence used to report Indels, included in Table S2. In red, TA1 sequence (AGAUUA); in green, TA2 sequence (UUAAA); in blue, TA3 sequence (UAUUA); in purple, TA4 sequence (CAUAAAUU); in grey, DR1 sequence (UUCACCUCUGC); in orange, TA23 sequence (UUAAAUAUUA).

The most common alteration of a TA sequence observed among patients showing Indels in that region was the fusion of TA2 and TA3 sequences due to a Del8nt between nt 1763 and 1770 (ID: 51) (Table 4). This Del8nt was among the most prevalent in the 50 patients included, with a relatively high median percentage of reads compared to the other Indels identified in more than 10% of patients (Table 2). Interestingly, the fusion of TA2 + TA3 due to Del 1763–1770 created the sequence TTAAATATTA. This sequence was coincident with that of a canonical/true TATA box [33], as the TA4, which we named TA23. Similar to the TA4 sequence, when viral genome double-stranded DNA is separated into single strands, (e.g., during cccDNA transcription), TA23 was found to be completely unpaired (Figure 3). In addition, the Del 1763–1770 was also present in Indel combinations identified in single patients. In Indels ID: 80, 81, 87, 90, and 100, this Del8nt was linked to an additional Ins around or inside DR1, and in ID: 24 to a big Ins at CURS. This last Indel caused the appearance of a premature stop codon in position 144, while the remaining Indels showing the Del 1763–1770 were associated with the prevalent altered *HBX* stop codon at position 135. However, it should be noted that this Del8nt was not strictly associated with the stop codon at position 135, as shown in a single HPL with the Del ID: 51, which showed the *HBX* stop codon at position 122 instead of 135 (Table S4).

### 3.5. Confirmation of Indel Variants by Cloning

Four patients with important percentages of variants with Indels by NGS were selected for cloning/sequencing analysis. The results of this additional analysis are shown in Table 5. In patient 2, cloning/sequencing confirmed the presence of the major complex Indel combination ID: 12 in a similar percentage to that found using NGS. In addition, cloning analysis also showed the presence of minor Indels such as the Ins ID: 74, and complex Indel combinations not identified by NGS. The presence of Ins 1825T (ID: 84) and Del 1825T (ID: 85), both highly prevalent in the included patients, was confirmed in patients 17 and 39, and in patient 20, respectively. Of note, the percentages of reads and clones that did not show any Indels were similar (indicated with a—in the Deletions and Insertions columns in Table 5).

### 3.6. Clinical Outcome of Patients Studied

Development of negative clinical outcomes associated with CHB was assessed in each of the 50 patients included for a median of 9.4 (IQR 8.7–12) years after obtaining selected samples. During this time, all patients received antiviral treatment, and only the five patients with advanced fibrosis (Ishak score > F3) on enrolment in the study developed negative clinical outcomes. One of these progressed to cirrhosis after superinfection with HCV 6.3 years after the sample analyzed was obtained and was excluded from further analysis. Two of the remaining four cases progressed to cirrhosis 3.9 and 3 years after the sample analyzed was obtained, respectively. The other two cases progressed to HCC 9.5 and 10.2 years after the sample analyzed was obtained, respectively; none of them showed cirrhosis at the time of HCC diagnosis.

All four patients showed variants encoding HBxCtermTrunc. Interestingly, a patient who progressed to cirrhosis and another who progressed to HCC, showed the Ins ID: 37 (Ins 1739G) and 38 (Ins 1746G/T), causing the highly prevalent stop codon at position 128 (Table 3). Notably, both patients showed higher percentages of reads and HPL harboring this stop codon than the median of all 50 patients, especially the patient who developed HCC: 0.9% vs. 1.5% vs. 0.4% of reads; and 20% vs. 33.3% vs. 8.3% of HPL in the cirrhotic, the HCC and median of all patients, respectively. The other patient who progressed to HCC showed the single nt Del ID: 36 (Del 1727), leading to the also prevalent *HBX* stop codon at position 129 (Table 3). Again, this patient showed higher percentages of reads and HPL harboring this stop codon than the median of all 50 patients: 1% vs. 0.5% of reads and 12.5% vs. 7.1%, respectively. The second patient who progressed to cirrhosis did not show any HPL with Indels, but one of them encoded a HBxCtermTrunc ending at codon 148.

## 4. Discussion

Indels in the *HBX* 3′ end region may lead to severe alterations in the HBx C-terminal end. Variants with Dels in that region and HBxCtermTrunc have been typically linked to HCC carcinogenesis [11,12]. However, the NGS analysis of the HBV quasispecies identified variants with 103 different Indels through the *HBX* 3′ end region in almost all (94%) of the 50 patients without HCC included in our study, most of them with no significant liver fibrosis. Previous studies by clone sequencing [16,17] and NGS [34] including CHB patients without HCC support these results. Peng et al. [16], reported 42 different variants with Indels in the Cp quasispecies, virtually all of them affecting *HBX*, in all 12 untreated HBeAg-positive CHB patients included. Hao et al. [17] characterized the Indels along the full-length HBV genome of 30 HBeAg-positive untreated CHB patients, of which 33/125 (26.4%) Dels and 14/45 (31.1%) Ins were within the *HBX* 3′ end region encoding aa 75–154 which we analyzed in this study. Li et al. [34] used NGS to analyze genome-wide mutation profiles, including deletion patterns, in 17 patients with advanced liver disease and 30 chronic HBV carriers. Interestingly, despite the younger age and absence of severe liver disease in the chronic carrier group, 11/21 (52%) Del validated in that group were in *HBX* 3′ end, while none of the patients with the advanced liver disease showed Dels in this region. Notably, the cut-off to eliminate technical artifacts adopted in that study was 1% in quasispecies, while in our study, it was 0.1% (as long as the sequence was present in both forward and reverse strands), thanks to the adaptation of our previously-described bioinformatics algorithm [19,20]. Additionally, the average sequencing coverage was 2047× and 687× in advanced liver disease and chronic carrier patients, respectively, while in our study, we obtained a median of 16735 reads [IQR, 9277–24,668]. The lower cut-off to eliminate technical artifacts along with higher sequencing coverages may explain why we detected a higher variability of Indels in the *HBX* 3′ end, even taking into account only single Dels (46 of 103 Indels identified in our study vs. 21 Dels identified in the study by Li et al.). Moreover, cloning/sequencing analysis of samples from four patients with important percentages of variants with Indels by NGS confirmed the presence of some Indels identified by NGS in their quasispecies. Importantly, this analysis confirmed the existence of Ins and Del around and inside the five T homopolymer between positions 1821 and 1825 identified by NGS (ID: 74, 84, 85, and 89), a region where the UDPS technology may introduce deletion and insertion sequencing errors [35]. Cloning/sequencing even confirmed the complex combination of Ins and Dels ID: 12. Altogether, suggests that Indel variants are usually present in HBV quasispecies, even in CHB patients without HCC.

HBx (only 154 aa long) is characterized by an astonishing pleiotropic function, which mainly relies on its interactions with cellular proteins that take part in HBV replication, transactivation, signaling pathway, protein degradation, cell death, and cell cycle [9]. In this regard, HBx C-terminal end contains sequence motifs essential for its transactivating activity [10], (i.e., protein–protein interactions), which may be affected by Indels identified in this study. In fact, almost all Indels identified altered the WT *HBX* stop codon, giving rise to variants encoding 44 different HBxCtermTrunc or HBxLong, making it reasonable to speculate that at least some of those Indels may develop different functions to WT HBx. For instance, we identified Indel variants potentially encoding different HBx + core fusion proteins, which would include almost the entire HBx and pre-core/core aa sequences, ending at 7 putative *HBX* stop codons between codons 355 to 363. These stop codons were associated with 15 different single or combined Indels between nt positions 1805 and 1843, detected in half of the patients included in this study. The fusion protein encoded by variants with these Indels may be relevant for the HBV replicative cycle: HBx is essential for initiating and maintaining transcription from cccDNA templates in de novo infected cells [36], but it is not clear how the nascent cccDNA can acquire this protein if the HBx mRNA is not able to be transcribed from this episome. This apparent contradiction may be explained by the existence of HBx forms with fused core aa sequences, which suggests that the resulting protein may be a “traveler HBx” form which, hypothetically, would reach infected cells to supply them with HBx. In support of this hypothesis, HBx has been detected in the serum of hepatitis B patients using ELISA [37], and HBx reactive determinants have been described in liver-derived HBcAg particles from human HBV and other hepadnaviruses [38]. However, it is important to note that the relevance to HBV infection and pathogenesis of this theoretical “traveler HBx” form is yet to be confirmed experimentally.

Additionally, in support of this hypothesis, Kim et al. [39] described the in vitro expression of a 40 KDa protein resulting from the fusion of *HBX* + core ORFs, which showed transactivating activity. Interestingly, the expression of this protein revealed the Ins 1821T, within the five T region where it has been described as highly prevalent Indels among our patients, Ins 1825T (ID: 84, observed in 38% of our patients), and Del 1825T (ID: 85, observed in 20% of our patients). It is likely that this T homopolymeric region facilitates polymerase slipping as a possible event responsible for Indels in this position, which would explain the high prevalence of Indels in position 1825, which were also reported in CHB patients quasispecies by clone sequencing [16,17]. The Ins 1825T is very often associated with the putative codon 362, which was indeed very common among our patients (observed in 48%) and represented a relatively high percentage of their reads compared to other stop codons. Indels in this five T homopolymeric region shift ORF from *HBX* to the core and continue translation until the stop codon of this last ORF, the stop codon 362, which may be an alternative mechanism similar to the frameshifts produced by PRF signals. Interestingly, the polyT homopolymeric sequence is highly conserved among other hepadnaviruses, and its predicted RNA folding is similar to that of HIV-1 PRF. For this reason, we considered that this polyT region might also be a potential HBV PRF signal, which may act as a mechanism to produce HBx + core fusion proteins, in addition to Ins in that region.

Another group of Indels that may play a functional role in the HBV replication cycle are those located in the TA region. We identified 39 Indels located between nt 1751 and 1787, many of which affected TA2 and TA3 sequences of the BCP, which are required for the optimal transcription of pcRNA [6]. Although these data showed high variability in the TA region, TA4 was completely preserved, suggesting a more essential role for this TA than the remaining ones. Previous studies performed by population (Sanger) sequencing [40,41] described frequent Del affecting TA1 to TA3 sequences showing two patterns: around 8 to 10, and around 19–21 Del, very similar to patterns observed in this study, as shown in Table 4, thus further supporting our results. Most of the Indels identified in the TA region led to HBxCtermTrunc and, interestingly, approximately a third of them were associated with a premature *HBX* stop codon at position 135, which has been identified in 28% of the 50 patients included. This premature stop codon was principally (but not exclusively) associated with the Del8nt between positions 1757 and 1773, among which Del8nt 1763–1770 (ID: 51) stands out, identified in 20% of patients studied. Interestingly, ID: 51 caused the fusion of TA2 and TA3 sequences, creating a sequence motif that coincided with that of a true (canonical) TATA box [33], which we named TA23. The structural prediction of this putative new TA revealed a complete unpairing, similar to the other true or canonical TA box in the BCP, TA4, and it maintains the same distance as TA3 to the pcRNA start sites, while it is located around 40 nt upstream of the pgRNA starting point [6]. Although this distance is slightly larger than the optimal TATA box position for achieving high tissue specificity (31 and 30 nt upstream of the RNA transcription starting site), the liver tissue-specific expression of pgRNA from TA23 would still be feasible [42]. This potential role as a true TA would explain the 1.8-fold increase in Cp activity relative to WT observed by Peng et al. [16] with TA1 deleted + Del8nt 1763–1770. Moreover, despite its association with HBxCtermTrunc, none of the patients who showed this Del8nt in our study experienced negative clinical outcomes. In previous studies, the Del 1763–1770 was identified in the HBV quasispecies of patients who progressed to cirrhosis or end-stage liver disease and patients who did not [15]. Thus, ID:51 did not appear to be associated with severe liver disease per se.

In the TA region, our attention was also drawn to the 14 complex Indel combinations containing Dels between nt 1756 and 1787 (affecting TA2 and TA3 sequences) and large Ins (24–28 nt) at CURS. Several of those variants showed a duplication in the sequence containing the α box motif (nts 1646–1668) and binding sites for C/EBPα (BCP positive regulatory motifs located in the CURS) [6,32]. This duplication also caused a drastic change in the HBx Hbox sequence, which may affect its predicted α helix structure [27], which would, in turn, hamper or eliminate the essential HBx-DDB1 interaction. DDB1 is a linker protein for the assembly of a large number of proteins to Cullin 4 (CUL4)-based E3 ubiquitin ligase [43]. The interaction of HBx with DDB1-CUL4-ROC1 (CRL4) E3 ligase is critical for ubiquitination and degradation of structural maintenance chromosome complex proteins 5 and 6 (Smc5/6), which antagonize HBV replication [31]. It would thus be logical to think that HPL with these Indels would be negatively selected. In fact, we found them in only four (8%) of the fifty patients studied; however, in one of them (patient 2), HPL with those complex Indel combinations represented the master sequence (more than 50% of reads obtained showed Ins: 1647 TCTTACATAAGAGGACTCTTGGAC Del: 1627 + 1758 − 1777, ID: 12) and their presence in the quasispecies of that patient was confirmed by cloning and sequencing. In addition, variants with duplications in the α box were reported earlier using cloning and sequencing studies [14,16]. This suggests a mechanism of these variants to initiate and maintain a productive HBV infection, an alternative to overcoming the Smc5/6 inhibitory effects. A possible explanation may be that expression of HBV cccDNA may be favored by the duplication of the C/EBPα binding site. This de novo C/EBP target may increase transcriptional activity, compensating for the decrease in HBV replication by alterations of the HBx coding sequence, which potentially impair HBx-DDB1 interaction.

The vast majority of the 103 Indels identified in this study altered the *HBX* stop codon, yielding a total of 44 different stop codons, 23 (52.3%) of which caused HBxCtermTrunc, and 9 (39.1%) of them were identified in more than 10% of patients. The truncated HBx forms have been reported to lose their transcriptional activity and their inhibitory effects on cell proliferation and transformation, as well as to enhance metastasis compared to full-length HBx, thus playing a critical role in HCC carcinogenesis [12,44,45]. However, in our patient cohort, only five patients experienced negative clinical outcomes after a median follow-up of 9.4 (IQR, 8.7–12) years, and this outcome may be associated solely with CHB infection in only four cases. Notably, three of them showed HPL with Ins ID: 36, 37, and 38, associated with *HBX* stop codons at positions 128 and 129. Interestingly, these three patients showed higher percentages of reads and HPL with these stop codons than the median reads and HPL/patient. It thus seems possible that the proportions of Indel variants encoding HBxCtermTrunc at positions 128 and 129 in the HBV quasispecies, rather than their mere presence, may contribute to their pathological effects. However, it was not possible to further explore this hypothesis in our study, as too few of the patients included experienced negative outcomes to allow for reliable statistical comparisons with those who experienced benign outcomes. In addition, we were not able to assess percentages of variants with Indels in the quasispecies of those four patients in serum samples obtained closer in time to a diagnosis of cirrhosis or HCC, as these additional serum samples were not available to us.

So far, we have discussed some possible functional roles of some of the 103 Indels identified in 94% of 50 patients analyzed. In this cohort of patients, we found that variants showing Indels were usually present as minor ones (median, 3.4% [IQR, 1.3–8.4%] of reads in each sample), and their median percentages were significantly higher in HBeAg-positive patients than in HBeAg-negative patients. However, when assessing individual Indels, due to disparities in the number of HBeAg-positive vs. HBeAg-negative patients (17 vs. 33), we found no significant differences between those groups. In fact, it must be remembered that this study is essentially descriptive, and further studies with larger and more balanced groups of patients, as well as in vitro phenotypic analyses, are required to confirm the functional roles of the Indels identified. For instance, the association of Dels between nt 1805 and 1843, and Ins within or very close to the five poly-T region between nt 1821–1825, with putative stop codons between positions 355 to 363 should be confirmed with longer NGS reads, encompassing at least the entire 3′ *HBX* and pre-core/core ORFs. In this regard, it would be ideal to provide confirmation using a third-generation NGS platform, applying error-correction procedures [35]. It would also be interesting to determine whether variants with combinations of Dels in the TA region and big Ins at CURS, such as ID: 12, with a highly altered HBx sequence, are able to sustain HBV replication, and perform additional studies assessing the effects over HBV replication and Cp activity of the new TA23 sequence motif due to the prevalent Del8nt 1763–1770 (ID: 51). Nevertheless, it seems reasonable to hypothesize that Indel events may be used as a strategy for increasing the coding capacity of the *HBX* 3′ end. Multiple examples exist of smart mechanisms for synthesizing new proteins from single genomic sequences in RNA viruses, which allow them to maximize genomic information content with a limited genome size. For instance, in bovine viral diarrhea virus, a 27-nt Ins in the NS2 protein-coding region has been associated with the cytopathogenic form of this virus [46], and a 15-nt Del in NS gene of H5N1 subtype avian influenza virus was associated with increased virulence of this subtype in chickens and mice [47]. However, Indel events are not necessarily associated with increases in virulence, as in the case of the SARS-CoV-2 spike gene, where minor quasispecies variants with an accumulation of Dels upstream and very close to the S1/S2 cleavage site have been associated with mild COVID-19 [48]. Thus, viruses can use Indels as mechanisms to encode alternative proteins, with important functional roles in the natural history of their infections. In line with this, our results suggest that at least some of the Indels that we identified in the quasispecies of the *HBX* 3′ end, may be linked to functional roles in the HBV replicative cycle. This suggests a general *HBX* multicoding mechanism, which would enable the characteristic HBx pleiotropic function.

## 5. Conclusions

In summary, the common presence in HBV quasispecies of HPL, equivalent to variants, with Indels in the 3′ end of *HBX* in CHB patients with nonsevere liver disease, suggests that these variants are “normal members”. Such Indels usually result in modification of the *HBX* stop codon, giving rise to truncated or long putative HBx versions with possible functional roles in the HBV replicative cycle. We also hypothesize that some of those HBx versions associated with Indels may be linked to severe progression in the case of high proportions. In addition, these Indels may also have consequences for transcription regulation of this virus due to sequence overlapping with regulatory elements such as Cp, ENH II, etc. Therefore, the phenotypical effects of these variants with Indels deserve further study. Altogether, this suggests that the production of 3′ *HBX* Indels is the origin of an HBx multicoding mechanism to increase the coding capacity for the small HBV genome and may be important for the extremely complex multifunctional activity of the HBx protein.

## Figures and Tables

**Table 1:** Characteristics of patients on obtaining the sample analyzed.

	Patients (*N* = 50)
Age (median years, IQR)	43 (35–55)
Gender (*N* Male, %)	42 (84)
HBV-DNA (median logIU/mL, IQR)	5.9 (5.3–7.9)
HBV genotype A (*N*, %) D (*N*, %) F (*N*, %)	
	39 (78)
	8 (16)
	3 (6)
HBeAg-negative (*N*, %)	33 (66)
ALT (median IU/L, IQR)	80 (57–115)
Liver fibrosis ^1^ ≤F3 (%) >F3 (%)	
	45 (90)
	5 (10)
Treatment history Previous NA (*N*, %) Previous IFN (*N*, %)	
	17 (34)
	2 (4)

^1^ Ishak fibrosis stage (F), assessed by liver biopsy. Abbreviations: N indicates number; IQR, interquartile range; HBV, hepatitis B virus; IU, international units; HBeAg, hepatitis B e antigen; ALT, alanine aminotransferase; NA, nucleoside/nucleotide analogs; IFN, interferon.

**Table 2:** Description and frequencies of reads and haplotypes of the insertions, deletions or combinations of both, identified in more than 10% of the 50 patients studied.

ID	Deletions	Insertions	N Patients (%)	Median % Reads/Patient (IQR)	Median % HPL/Patient (IQR)
11	1646	-	6 (12)	0.5 (0.4–0.6)	6.5 (2.8–10.4)
30	1692	1697TT	6 (12)	0.5 (0.4–0.6)	7.5 (6.1–14.6)
37	-	1739G	9 (18)	0.3 (0.3–0.5)	8.3 (7.1–14.3)
38	-	1746G/T	7 (14)	0.4 (0.3–0.4)	10 (6.7–12.7)
40	1749	-	7 (14)	0.4 (0.3–0.5)	6.7 (5.2–11.3)
51	1763–1770	-	10 (20)	1.7 (0.9–2.1)	5.5 (3.0–8.2)
59	-	1781C	5 (10)	0.6 (0.6–0.7)	8.3 (4.8–8.3)
74	-	1820C	7 (14)	0.4 (0.4–0.9)	4.3 (1.9–4.8)
84	-	1825T	19 (38)	1.5 (0.7–2.1)	4.8 (2.8–6.9)
85	1825	-	10 (20)	0.4 (0.4–0.8)	4.1 (2.8–5.1)
88	-	1826C/T	9 (18)	0.6 (0.4–0.9)	4.8 (2.6–8.3)
103	-	1838A	5 (10)	1.2 (0.9–2.7)	2.3 (2.2–5.9)

Abbreviations: ID indicates code to identify single insertion or deletion, or combinations of them (see Table S3); N, number; IQR, interquartile range; HPL, haplotypes.

**Table 3:** Description and frequencies of reads and haplotypes of the altered stop codons identified in more than 10% of the 50 patients studied.

*HBX* Stop Codon	IDs	N Patients (%)	Median % Reads/Patient (IQR)	Median % HPL/Patient (IQR)
95	2, 3, 7, 8	5 (10)	0.4 (0.3–0.5)	5.9 (5.3–8.3)
109	28, 29, 30, NA	9 (18)	0.5 (0.4–0.6)	6.7 (4.3–16.7)
125	18, NA	8 (16)	1.9 (0.6–3.8)	3.8 (2.2–5.8)
128	37, 38, 41	15 (30)	0.4 (0.3–0.6)	8.3 (5.6–18.3)
129	6, 11, 23, 25, 34, 35, 36, 39, 40	21 (42)	0.5 (0.4–0.6)	7.1 (4.8–11.1)
132	45, 46, 49, 57	5 (10)	0.5 (0.5–0.8)	2.6 (2.6–4.3)
135	14, 15, 22, 47, 48, 51, 53, 55, 80, 81, 87, 90, 100	14 (28)	2.0 (0.5–4.9)	6.1 (3.0–11.8)
138	59	5 (10)	0.6 (0.6–0.7)	8.3 (4.8–8.3)
149	63, 72	5 (10)	0.7 (0.4–1.0)	4.5 (4.2–4.8)
156	12, 13, 27, 58, 76, 82, 89	7 (14)	2.6 (0.7–7.3)	7.1 (5.5–7.7)
179	85	10 (20)	0.4 (0.4–0.8)	4.1 (2.8–5.1)
180 *	75, 86, 98	5 (10)	0.9 (0.6–1.2)	5.1 (3.6–5.9)
207 *	101, NA	6 (12)	0.5 (0.4–0.6)	3 (2.4–4.4)
360 *	70, 92	5 (10)	0.6 (0.6–1.1)	2.6 (2.3–4.3)
362 *	74, 78, 84, 88	24 (48)	1.6 (0.8–2.1)	5.8 (3.1–10.7)

Abbreviations: *HBX* indicates hepatitis B X open reading frame; IDs, code to identify single insertion or deletion, or combinations of them, described in Table S3; N, number; IQR, interquartile range; HPL, haplotypes; NA, haplotype/s without insertions and/or deletions. * Putative stop codons of the HPL without stop codon in *HBX*, obtained by extension of those HPL with reference sequences V01460 (for genotype D HPL) and X02763 (for genotype A HPL) and continue translation.

**Table 4:** Alterations in the core promoter TATA-like boxes caused by insertions and deletions identified between nucleotides 1751 and 1787 in the 50 patients studied.

TA Alteration	Cause of Alteration and Indels Involved	N Patients (%)	Median % Reads/Patient (IQR)	Median % HPL/Patient (IQR)
Insertion in TA1 (nt 1750–1755)	**Ins:** ID: 41 (Ins 1751G)	3 (6)	0.3–0.4 *	3.4–5.9 *
Partial or total TA1 or TA2 (nt 1758–1762) elimination	7 to 10 nt Del between nt 1754 and 1767: **Dels:** ID: 42, 45, 47, 48, 49, 50 **Ins + Del**: ID: 16	5 (10)	0.5 (0.4–2.0)	3.6 (2.2–4.5)
Partial or total TA2 + TA3 (nt 1758–1775) elimination	Dels between nt 1756 and 1787: **Dels**: ID: 43, 44, 46 **Ins + Dels:** ID: 9, 12, 13, 14, 15, 17, 18, 19, 20, 22, 26, 27	3 (6)	0.9–71.2 *	2.3–61.8 *
TA2 + TA3 Fusion	8 nt Del between nt 1763 and 1770: **Del:** ID: 51 **Ins + Dels:** ID: 24, 80, 81, 87, 90, 100	10 (20)	1.8 (0.9–4.3)	5.5 (3.0–17.5)
Partial or total TA3 (nt 1771–1775) elimination	8 to 10 nt Del between nt 1763 and 1776 **Dels:** ID: 52, 53, 55, 56, 57	7 (14)	0.4 (0.4–2.1)	2.6 (2.4–5.6)
No TA affected	**Ins:** ID: 58 (Ins 1768GTT/ATT), 59 (Ins 1781C), 60 (Ins 1785C) **Del**: ID: 54 (Del 1766)	10 (20)	0.6 (0.4–0.9)	6.5 (4.3–8.3)

Abbreviations: TA indicates TATA-like boxes; N, number; IQR, interquartile range; HPL, haplotypes; nt, nucleotides; Ins, insertions; Del, deletions; IDs, code to identify single Ins or Del, or combinations of them, described in Table S4. * No median was calculated due to low number of patients, instead the maximum and minimum percentage of reads and haplotypes per patient are shown.

**Table 5:** Description of the insertions, deletions, or combinations of both, identified by cloning/sequencing analysis in the 4 patients selected.

Patient (N Clones Analyzed)	Deletions	Insertions	N Clones (%)	ID	N Reads (%)
2 (24)	1627 + 1758 − 1777	1647 TCTTACATAAGAGGACTCTTGGAC	12 (50)	12	9554 (51.9)
	-	1820 C	1 (4.2)	74	75 (0.4)
	1627 + 1726 + 1758 − 1777	1647 TCTTACATAAGAGGACTCTTGGAC	2 (8)	-	-
	1627 + 1758 − 1777	1647 TCTTACATAAGAGGACTCTTGGAC + 1822 ATTCAA + 1825 T	1 (4.2)	-	-
	1627	1600T + 1647 TCTTACATAAGAGGACTCTTGGAC	1 (4.2)	-	-
	-	-	7 (29.2)	-	4692 (25.5)
17 (29)	-	1825 T	2 (6.9)	84	3140 (14.2)
	-	1909 TG	1 (3.4)	*	*
	-	-	26 (89.7)	-	18736 (84.8)
20 (18)	1825	-	1 (5.6)	85	3011 (15.5)
	-	1826 TTC	6 (33.3)	89	2171 (11.2)
	-	-	11 (61)	-	14064 (72.5)
39 (19)	-	1605 T	1 (5.3)	-	-
	-	1825 T	7 (36.8)	84	3242 (22.9)
	-	1895 T	1 (5.3)	*	*
	-	-	10 (52.6)	-	9701 (68.5)

Abbreviations: N Clones indicates number of clones showing a specific insertion, deletion or combination of both; ID, code to designate single insertion or deletion, or combinations of them identified by next-generation sequencing (see Table S3); N Reads, number of next-generation sequencing reads showing a specific single insertion or deletion, or a combination of both. * Insertions that have not been assessed in next-generation sequencing reads since they are located out of hepatitis B X gene open reading frame.

## Data Availability

The next-generation sequencing raw data presented in this study are openly available in NCBI’s Sequence Read Archive (SRA), at BioProject reference number PRJNA625435. BioSample reference numbers are provided in Table S1.

## Supplementary Materials

The following supporting information can be downloaded at: https://www.mdpi.com/article/10.3390/biomedicines10051194/s1. Table S1. Biosample accession numbers of next-generation sequencing raw data. Table S2. Reference sequences of hepatitis B virus genotypes A, D/E and F used for alignining forward and reverse haplotypes of each sample, with abundances not below 0.1%. Table S3. Description of 103 different single insertions (Ins) or deletions (Del), or combinations of them that affected the HBx coding region, identified in the 50 patients studied. Table S4. Description of 39 different single insertions (Ins) or deletions (Del), or combinations of them that affected the region encompassing the four TATA-like boxes (TA) of the core promoter, between nucleotides 1751 and 1787.

## References

[1] World Health Organization Hepatitis B. https://www.who.int/news-room/fact-sheets/detail/hepatitis-b.

[2] Martinez M.G., Testoni B., Zoulim F. (2019). Biological Basis for Functional Cure of Chronic Hepatitis B. J. Viral Hepat..

[3] Nassal M. (2015). HBV CccDNA: Viral Persistence Reservoir and Key Obstacle for a Cure of Chronic Hepatitis B. Gut.

[4] Tu T., Budzinska M.A., Shackel N.A., Urban S. (2017). HBV DNA Integration: Molecular Mechanisms and Clinical Implications. Viruses.

[5] Panjaworayan N., Roessner S.K., Firth A.E., Brown C.M. (2007). HBVRegDB: Annotation, Comparison, Detection and Visualization of Regulatory Elements in Hepatitis B Virus Sequences. Virol. J..

[6] Quarleri J. (2014). Core Promoter: A Critical Region Where the Hepatitis B Virus Makes Decisions. World J. Gastroenterol..

[7] Kramvis A., Kew M.C. (1999). The Core Promoter of Hepatitis B Virus. J. Viral Hepat..

[8] Levrero M., Zucman-Rossi J. (2016). Mechanisms of HBV-Induced Hepatocellular Carcinoma. J. Hepatol..

[9] Xie N., Chen X., Zhang T., Liu B., Huang C. (2014). Using Proteomics to Identify the HBx Interactome in Hepatitis B Virus: How Can This Inform the Clinic?. Expert Rev. Proteom..

[10] Kumar V., Jayasuryan N., Kumar R. (1996). A Truncated Mutant (Residues 58–140) of the Hepatitis B Virus X Protein Retains Transactivation Function. Proc. Natl. Acad. Sci. USA.

[11] Zhang A.Y., Lai C.L., Poon R.T.P., Huang F.Y., Seto W.K., Fung J., Wong D.K.H., Yuen M.F. (2016). Hepatitis B Virus Full-Length Genomic Mutations and Quasispecies in Hepatocellular Carcinoma. J. Gastroenterol. Hepatol..

[12] Ning-Fang M., Lau S.H., Hu L., Xie D., Wu J., Yang J., Wang Y., Wu M.C., Fung J., Bai X. (2008). COOH-Terminal Truncated HBV X Protein Plays Key Role in Hepatocarcinogenesis. Clin. Cancer Res..

[13] Rodriguez-Frias F., Buti M., Tabernero D., Homs M. (2013). Quasispecies Structure, Cornerstone of Hepatitis B Virus Infection: Mass Sequencing Approach. World J. Gastroenterol..

[14] Günther S., Piwon N., Iwanska A., Schilling R., Meisel H., Will H. (1996). Type, Prevalence, and Significance of Core Promoter/Enhancer II Mutations in Hepatitis B Viruses from Immunosuppressed Patients with Severe Liver Disease. J. Virol..

[15] Preikschat P., Günther S., Reinhold S., Will H., Budde K., Neumayer H.H., Krüger D.H., Meisel H. (2002). Complex HBV Populations with Mutations in Core Promoter, C Gene, and Pre-S Region Are Associated with Development of Cirrhosis in Long-Term Renal Transplant Recipients. Hepatology.

[16] Peng Y., Liu B., Hou J., Sun J., Hao R., Xiang K., Yan L., Zhang J., Zhuang H., Li T. (2015). Naturally Occurring Deletions/Insertions in HBV Core Promoter Tend to Decrease in Hepatitis B e Antigen-Positive Chronic Hepatitis B Patients during Antiviral Therapy. Antivir. Ther..

[17] Hao R., Xiang K., Peng Y., Hou J., Sun J., Li Y., Su M., Yan L., Zhuang H., Li T. (2015). Naturally Occurring Deletion/Insertion Mutations within HBV Whole Genome Sequences in HBeAg-Positive Chronic Hepatitis B Patients Are Correlated with Baseline Serum HBsAg and HBeAg Levels and Might Predict a Shorter Interval to HBeAg Loss and Seroconversi. Infect. Genet. Evol..

[18] Caballero A., Gregori J., Homs M., Tabernero D., Gonzalez C., Quer J., Blasi M., Casillas R., Nieto L., Riveiro-Barciela M. (2015). Complex Genotype Mixtures Analyzed by Deep Sequencing in Two Different Regions of Hepatitis B Virus. PLoS ONE.

[19] Ramírez C., Gregori J., Buti M., Tabernero D., Camós S., Casillas R., Quer J., Esteban R., Homs M., Rodríguez-Frías F. (2013). A Comparative Study of Ultra-Deep Pyrosequencing and Cloning to Quantitatively Analyze the Viral Quasispecies Using Hepatitis B Virus Infection as a Model. Antivir. Res..

[20] Gregori J., Esteban J.I., Cubero M., Garcia-Cehic D., Perales C., Casillas R., Alvarez-Tejado M., Rodríguez-Frías F., Guardia J., Domingo E. (2013). Ultra-Deep Pyrosequencing (UDPS) Data Treatment to Study Amplicon HCV Minor Variants. PLoS ONE.

[21] Edgar R.C., Drive R.M., Valley M. (2004). MUSCLE: Multiple Sequence Alignment with High Accuracy and High Throughput. Nucleic Acids Res..

[22] R Core Team (2013). R: A Language and Environment for Statistical Computing.

[23] Pages H., Aboyoun P., Gentleman R., DebRoy S. (2011). Biostrings: String Objects Representing Biological Sequences, and Matching Algorithms.

[24] Paradis E., Claude J., Strimmer K. (2004). APE: Analyses of Phylogenetics and Evolution in R Language. Bioinformatics.

[25] Gruber A.R., Lorenz R., Bernhart S.H., Neuböck R., Hofacker I.L. (2008). The Vienna RNA Websuite. Nucleic Acids Res..

[26] R Core Team (2021). A Language and Environment for Statistical Computing.

[27] Li T., Robert E.I., van Breugel P.C., Strubin M., Zheng N. (2010). A Promiscuous Alpha-Helical Motif Anchors Viral Hijackers and Substrate Receptors to the CUL4-DDB1 Ubiquitin Ligase Machinery. Nat. Struct. Mol. Biol..

[28] Firth A.E., Brierley I. (2012). Non-Canonical Translation in RNA Viruses. J. Gen. Virol..

[29] Mouzakis K.D., Lang A.L., Vander Meulen K.A., Easterday P.D., Butcher S.E. (2013). HIV-1 Frameshift Efficiency Is Primarily Determined by the Stability of Base Pairs Positioned at the MRNA Entrance Channel of the Ribosome. Nucleic Acids Res..

[30] Belew A.T., Meskauskas A., Musalgaonkar S., Adnavi V.M., Sulima S.O., Kasprzak W.K., Shapiro B.A., Dinman J. (2014). Ribosomal Frameshifting in the CCR5 MRNA Is Regulated by MiRNAs and the NMD Pathway. Nature.

[31] Murphy C.M., Xu Y., Li F., Nio K., Reszka-Blanco N., Li X., Wu Y., Yu Y., Xiong Y., Su L. (2016). Hepatitis B Virus X Protein Promotes Degradation of SMC5/6 to Enhance HBV Replication. Cell Rep..

[32] Choi B.H., Park G.T., Rho H.M. (1999). Interaction of Hepatitis B Viral X Protein and CCAAT/ Enhancer-Binding Protein Alpha Synergistically Activates the Hepatitis B Viral Enhancer II/Pregenomic Promoter. J. Biol. Chem..

[33] Lee T.Y., Chang W.C., Hsu J.B.K., Chang T.H., Shien D.M. (2012). GPMiner: An Integrated System for Mining Combinatorial Cis-Regulatory Elements in Mammalian Gene Group. Ser. Adv. Bioinform. Comput. Biol..

[34] Li F., Zhang D., Li Y., Jiang D., Luo S., Du N., Chen W., Deng L., Zeng C. (2015). Whole Genome Characterization of Hepatitis B Virus Quasispecies with Massively Parallel Pyrosequencing. Clin. Microbiol. Infect..

[35] Garcia-Garcia S., Cortese M.F., Rodríguez-Algarra F., Tabernero D., Rando-Segura A., Quer J., Buti M., Rodríguez-Frías F. (2021). Next-Generation Sequencing for the Diagnosis of Hepatitis B: Current Status and Future Prospects. Expert Rev. Mol. Diagn..

[36] Lucifora J., Arzberger S., Durantel D., Belloni L., Strubin M., Levrero M., Zoulim F., Hantz O., Protzer U. (2011). Hepatitis B Virus X Protein Is Essential to Initiate and Maintain Virus Replication after Infection. J. Hepatol..

[37] Pál J., Nyárády Z., Marczinovits I., Pár A., Ali Y.S., Berencsi G., Kvell K., Németh P. (2006). Comprehensive Regression Analysis of Hepatitis B Virus X Antigen Level and Anti-HBx Antibody Titer in the Sera of Patients with HBV Infection. Pathol. Oncol. Res..

[38] Feitelson M.A. (1986). Products of the “X” Gene in Hepatitis B and Related Viruses. Hepatology.

[39] Kim S.K., Jang S.K., Rho H.M. (1994). Effect of Frameshift Mutation in the Pre-C Region of Hepatitis B Virus on the X and C Genes. J. Gen. Virol..

[40] Deng H., Dong J., Cheng J., Huangfu K.J., Shi S.S., Hong Y., Ren X.M., Li L. (2002). Quasispecies Groups in the Core Promoter Region of Hepatitis B Virus. Hepatobiliary Pancreat. Dis. Int..

[41] Zhang D., Dong P., Zhang K., Deng L., Bach C., Chen W., Li F., Protzer U., Ding H., Zeng C. (2012). Whole Genome HBV Deletion Profiles and the Accumulation of PreS Deletion Mutant during Antiviral Treatment. BMC Microbiol..

[42] Ponjavic J., Lenhard B., Kai C., Kawai J., Carninci P., Hayashizaki Y., Sandelin A. (2006). Transcriptional and Structural Impact of TATA-Initiation Site Spacing in Mammalian Core Promoters. Genome Biol..

[43] Angers S., Li T., Yi X., MacCoss M.J., Moon R.T., Zheng N. (2006). Molecular Architecture and Assembly of the DDB1-CUL4A Ubiquitin Ligase Machinery. Nature.

[44] Li W., Li M., Liao D., Lu X., Gu X., Zhang Q. (2016). Carboxyl-Terminal Truncated HBx Contributes to Invasion and Metastasis via Deregulating Metastasis Suppressors in Hepatocellular Carcinoma. Oncotarget.

[45] Tu H., Bonura C., Giannini C., Mouly H., Soussan P., Kew M., Paterlini-Bréchot P., Bréchot C., Kremsdorf D. (2001). Biological Impact of Natural COOH-Terminal Deletions of Hepatitis B Virus X Protein in Hepatocellular Carcinoma Tissues. Cancer Res..

[46] Tautz N., Meyers G., Stark R., Dubovi E.J., Thiel H.J. (1996). Cytopathogenicity of a Pestivirus Correlates with a 27-Nucleotide Insertion. J. Virol..

[47] Long J.X., Peng D.X., Liu Y.L., Wu Y.T., Liu X.F. (2008). Virulence of H5N1 Avian Influenza Virus Enhanced by a 15-Nucleotide Deletion in the Viral Nonstructural Gene. Virus Genes.

[48] Andres C., Garcia-Cehic D., Gregori J., Piñana M., Rodriguez-Frias F., Guerrero-Murillo M., Esperalba J., Rando A., Goterris L., Codina M.G. (2020). Naturally Occurring SARS-CoV-2 Gene Deletions Close to the Spike S1/S2 Cleavage Site in the Viral Quasispecies of COVID19 Patients. Emerg. Microbes Infect..

